# Apixaban-Induced Pseudo-Ludwig’s Angina

**DOI:** 10.7759/cureus.26740

**Published:** 2022-07-11

**Authors:** Nicholas Figaro, Keegan Figaro, Rajeev V Seecheran, Valmiki K Seecheran, Stanley Giddings, Naveen A Seecheran

**Affiliations:** 1 Otolaryngology, Eric Williams Medical Sciences Complex, Mt. Hope, TTO; 2 Internal Medicine, Eric Williams Medical Sciences Complex, Mt. Hope, TTO; 3 Internal Medicine, University of Kansas Medical Center, Wichita, USA; 4 Internal Medicine, Eric Williams Medical Sciences Complex, Champs Fleurs, TTO; 5 Infectious Diseases, The University of the West Indies, St. Augustine, TTO; 6 Cardiology, The University of the West Indies, St. Augustine, TTO

**Keywords:** factor xa inhibitor, apixaban, direct oral anticoagulant (doac), pseudo-ludwig’s angina, ludwig’s angina

## Abstract

Ludwig’s angina describes fulminant cellulitis involving the oro- and hypopharynx, which typically stems from bacterial pathogens, whereas “pseudo-Ludwig’s angina” is ascribed to sublingual swelling due to noninfectious causes. There is a paucity of case reports implicating warfarin as the culprit for sublingual hematoma mimicking Ludwig’s angina; however, we describe a novel case of apixaban-induced pseudo-Ludwig’s angina, which was successfully managed with urgent surgical intervention and supportive care with antibiotic and glucocorticoid therapy.

## Introduction

Ludwig’s angina is severe submandibular cellulitis with secondary involvement of the sublingual and submental spaces, usually precipitated by a bacterial infection or penetrating injury [1].

Sublingual hematoma secondary to anticoagulant therapy, such as warfarin, has been labeled “pseudo-Ludwig’s angina” to differentiate it from the classic syndrome, albeit with similar dreaded and even fatal complications. Currently, there is a paucity of cases describing this exceedingly rare phenomenon since its initial description in 1976 [2].

Here, we describe a novel case of apixaban-induced pseudo-Ludwig’s angina, which, to the best of our knowledge, is probably reported for the first time in medical literature, and which was successfully managed with urgent surgical intervention and supportive care with antibiotic and glucocorticoid therapy.

## Case presentation

A 78-year-old South Asian male with a medical history of permanent atrial fibrillation, on apixaban 2.5 milligrams twice daily, and chronic kidney disease stage 3b (baseline serum creatinine of 1.6 mg/dL (normal: 0.5-1.0 mg/dL) and estimated glomerular filtration rate of 44 mL/min/1.73m2) presented to the emergency department with difficulty speaking and swallowing, consistent with trismus, worsening during the preceding 24 hours. He had no other pertinent medication usage, significant travel, or sick contact history. He also did not report any recent intraoral infection or dental injury.

**Figure 1:**
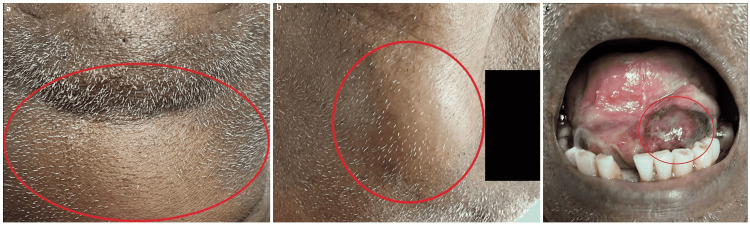
The patient’s physical examination. (A) The patient’s frontal neck swelling reflecting the fulminant cellulitis (encircled in red). (B) The patient’s right facial swelling reflecting the cellulitis extending to the submaxillary and submandibular spaces (encircled in red). (C) The patient’s suppurated, sublingual hematoma disseminated into fulminant Ludwig’s angina (encircled in red).

His vital signs indicated a systolic blood pressure of 105 mmHg, an irregular heart rate of 123 beats/minute, a respiratory rate of 20 breaths/minute, and he was afebrile with an oxygen saturation of 94% on ambient air. His physical examination was remarkable for his frail appearance. He had a bilateral lower face, jaw, neck, and intraoral swelling with an elevation of the tongue (bull-neck) and respiratory distress without stridor. There was a suppurative sublingual hematoma with surrounding inflammatory and necrotic changes (Figure 1). The patient also had irregular heart sounds, an indiscernible jugular venous pulse, and occasional scattered crackles with mild pitting edema consistent with clinical heart failure.

**Figure 2:**
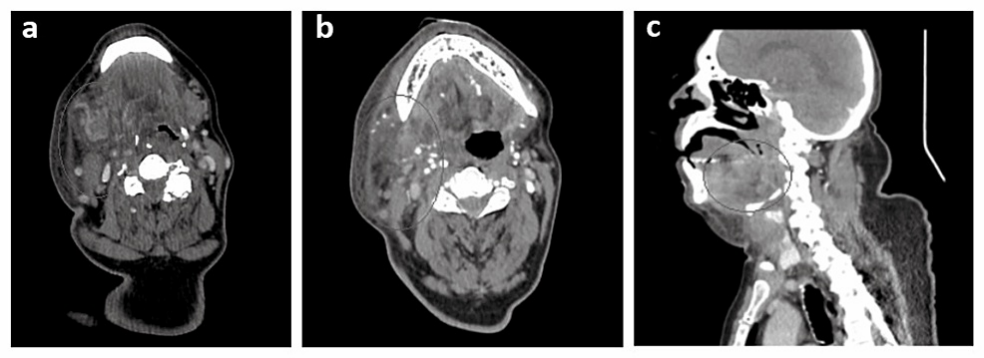
The patient’s computed tomography scan of the head and neck. (A) An axial image indicating asymmetrical right parapharyngeal enlargement causing effacement of the adjacent pharyngeal space (encircled in black). (B) An axial image indicating right submandibular and sublingual soft tissue swelling (encircled in black). (C) A sagittal image indicating significant submental swelling (encircled in black).

Severe acute respiratory syndrome coronavirus 2 (SARS-CoV-2) IgM and IgG antibodies serology (Abbott Laboratories, Chicago, IL) was negative upon arrival at the emergency department. A 12-lead electrocardiogram revealed atrial fibrillation with a rapid ventricular response (AF-RVR) and secondary ST-T changes. An emergent non-contrast computed tomography scan of the head, neck, and chest revealed a right parapharyngeal abscess with near-complete effacement of the oropharynx at the level of the epiglottis and subhyoid space (Figure 2). Pertinent diagnostic laboratory investigations included a leukocytosis of 14 x 10^3^/mm^3^ (normal: 4-10 x 10^3^/mm^3^) with a “neutrophilic left shift” and acute kidney injury (AKI) with a serum creatinine of 2.2 mg/dL (normal: 0.5-1.0 mg/dL) and troponin I of 0.24 ng/mL (normal < 0.15 ng/mL). A bedside two-dimensional transthoracic echocardiogram demonstrated a preserved left ventricular function with impaired diastolic function and mild pulmonary hypertension.

The patient was subsequently admitted to the intensive care unit and optimal medical therapy was initiated for the tentative diagnosis of fulminant Ludwig’s angina with septic shock and AF-RVR. His sequential organ failure assessment (SOFA) score was 11, which prognosticated a 50% inpatient mortality. He was immediately intubated by a senior consultant anesthesiologist and commenced on low-dose intravenous norepinephrine and amiodarone. In addition, he was initiated on broad-spectrum antibiotics with meropenem, linezolid, levofloxacin, metronidazole, and high-potency dexamethasone. His cardiovascular regimen of apixaban and bisoprolol was discontinued in the anticipation of imminent surgical intervention and progressive sepsis. Andexanet alfa (recombinant factor X) was not administered as a reversal agent due to regulatory and accessibility issues for emergency use.

**Figure 3:**
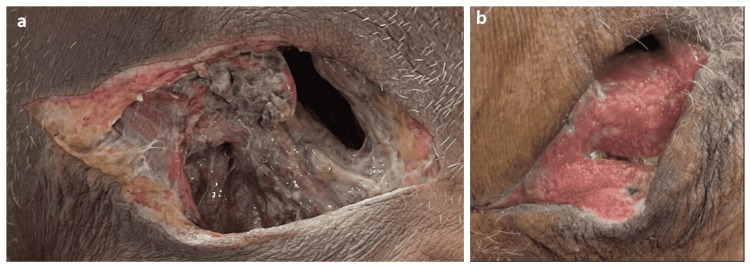
The patient’s surgical site for incision and drainage. (A) The index surgical site on admission indicating purulent, necrotic tissue. (B) The surgical site at discharge (21 days) indicating granulation with resolving infection.

The patient’s ensuing three-week hospitalization was tenuous after undergoing an urgent incision and drainage under general anesthesia 36 hours after admission (Figure 3). He also underwent an early tracheostomy and required emergency dialysis after developing refractory volume overload associated with anuria, and serum creatinine of 9 mg/dL consistent with AKI. Adjunctive therapies included mechanical ventilation via tracheostomy, nutritional and caloric replacement, antibiotics, and steroids, which were de-escalated as appropriate with physiotherapy sessions. Surgical wound cultures confirmed *Streptococcus viridans* species involvement.

The patient was eventually discharged and steadily improved where he no longer required the tracheostomy and intermittent hemodialysis. An interval surveillance transthoracic echocardiogram did not reveal any evidence of native valve endocarditis, and the patient's serum creatinine returned to baseline (1.6 mg/dL; normal: 0.5-1.0 mg/dL). He was resumed on his pre-existing cardiovascular regimen excluding apixaban during the interim period as his CHA2DS2-VASc (congestive heart failure, hypertension, age ≥ 75 years, diabetes mellitus, stroke or transient ischemic attack, vascular disease, age 65 to 74 years, sex category) and HAS-BLED (hypertension, abnormal renal/liver function, stroke, bleeding history or predisposition, labile international normalized ratio, elderly, drugs/alcohol concomitantly) scores were each 3 (3.2% risk of a cerebrovascular event and 5.8% risk of a major bleeding episode, respectively).

## Discussion

Ludwig’s angina describes fulminant cellulitis involving the oro- and hypopharynx, which typically stems from bacterial pathogens, whereas “pseudo-Ludwig’s angina” is ascribed to sublingual swelling due to noninfectious causes [2,3]. This eponymous syndrome is named after the German physician Wilhelm Frederick von Ludwig, who initially described it in 1836 [4]. The term “angina” is derived from the Latin word “angere,” which translates to “choke,” referring to the sensation of strangling secondary to obstruction of the airway [5].

The most common etiology of Ludwig’s angina is dental-related, accounting for nearly 90% of cases [6]. Systemic conditions such as diabetes mellitus and a compromised immune system predispose to Ludwig’s angina [6]. There are four pivotal aspects of Ludwig’s angina: (1) prompt airway management, (2) aggressive broad-spectrum antibiotic therapy, (3) incision and drainage for refractory disease, and (4) adequate nutrition and hydration support [6]. A paucity of case reports has implicated sublingual hematoma secondary to hemorrhage from warfarin for pseudo-Ludwig’s angina [7-9]. Angioedema and lingual carcinoma should be considered differential diagnoses [10]. The patient’s biopsy and culture confirmed it as a localized hematoma with disseminated viridans septicemia, which alludes to a “double-hit” phenomenon. This case is interesting with respect to several facets. Although the initial impression was a sublingual hematoma with a pseudo-Ludwig’s diagnosis, we speculate that this hematoma became secondarily infected and transitioned to traditional Ludwig’s angina with fulminant septic shock. We postulated that his sublingual hematoma resulted from his direct oral anticoagulant (DOAC) therapy, apixaban, and his relatively high risk of bleeding, as stratified by his HAS-BLED score of 3 and chronic kidney disease. To our knowledge, this case is unique as the hematoma was precipitated by this novel DOAC therapy and not because of warfarin, as in all previously reported cases.

Apixaban is a highly selective, orally bioavailable, and reversible direct inhibitor of free and clot-bound factor Xa [11]. It was approved for the prevention of stroke in patients with atrial fibrillation after the conclusion of the “Apixaban for Reduction in Stroke and Other Thromboembolic Events in Atrial Fibrillation” (ARISTOTLE) trial in 2011. It was superior to warfarin in reducing rates of stroke (annual incidence of 1.27% vs. 1.60%) and with less major bleeding (annual incidence of 2.13% vs. 3.09%) [12,13]. Unfortunately, andexanet alfa, the “reversal agent” for apixaban, which was approved in 2018, was not administered as aforementioned and may have been potentially lifesaving in this clinical scenario. It reverses the effect of all anticoagulants that act directly through factor Xa or by binding antithrombin III [14].

Airway management is a crucial intervention and is deemed a “primary concern” as its compromise is the leading cause of mortality in Ludwig’s angina [15,16]. Surgical incision and drainage are the mainstays in managing disseminated deep neck infections refractory to medical management [15]. With respect to our patient, there was a measured risk-benefit analysis of proceeding with immediate tracheostomy and debridement under general anesthesia to secure the airway versus a conservative approach in allowing the full dose of apixaban to be eliminated as no antidote was available. We opted for the latter approach as it was safer from a patient perspective of mitigating intraoperative bleeding without a safety net of readily available blood transfusion products in our limited resource setting. This strategy also allowed for the institution of glucocorticoid therapy, which may yield a marginal benefit without adversely affecting mortality [17]. In addition, it permitted the team to implement the Surviving Sepsis Campaign guidelines with respect to “golden hour” antibiotic timing, vasopressor management, and lung-protective mechanical ventilation [18].

## Conclusions

Apixaban can cause a sublingual hematoma, which can be infected secondarily leading to septicemia and sequential organ failure. Prompt recognition and an urgent multidisciplinary approach are life-saving in this potentially fatal condition.
